# Prevalence and Antimicrobial Resistance of Bacterial Uropathogens Isolated from Iranian Kidney Transplant Recipients: A Systematic Review and Meta-Analysis

**Published:** 2019-12

**Authors:** Abbas SHAPOURI MOGHADDAM, Maryam ARFAATABAR, Jalil TAVAKOL AFSHARI, Ali SHAKERIMOGHADDAM, Zahra MOHAMMADZAMANI, Azad KHALEDI

**Affiliations:** 1. Department of Immunology, BuAli Research Institute, Faculty of Medicine, Mashhad University of Medical Sciences, Mashhad, Iran; 2. Department of Medical Laboratory Sciences, Kashan Branch, Islamic Azad University, Kashan, Iran; 3. Infectious Diseases Research Center, Kashan University of Medical Sciences, Kashan, Iran; 4. Department of Microbiology and Immunology, School of Medicine, Kashan University of Medical Sciences, Kashan, Iran

**Keywords:** Kidney transplant, Urinary tract infection, Iran

## Abstract

**Background:**

Urinary tract infection (UTI) is a major complication in patients who receive the kidney transplant. We aimed to evaluate the prevalence and antimicrobial resistance of bacterial uropathogens isolated from Iranian kidney transplant recipients.

**Methods:**

We searched according to Prisma protocol for UTI infection, prevalence, occurrence and distribution of bacteria and their pattern of antibiotic resistance among Iranian patients who receive kidney transplant through online electronic databases with MeSh terms and text words in published references in both Persian and English languages during 1990–2017. Data analysis was performed using Comprehensive meta-analysis software (CMA) by Cochrane Q and I^2^ Random Effects Model.

**Results:**

Eleven studies met the eligible inclusion criteria. The prevalence of UTI among kidney transplant patients varied from 11.7% to 67.5%. The combined prevalence of UTI was 32.6%. Among Gram-negative pathogens causing UTI, *E. coli* was the most dominant followed by *Klebsiella pneumonia* with prevalence 41.3% and 11.9%, respectively. Also, amongst Gram-positive bacteria, the highest prevalence belonged to *Enterococcus* spp*.* (9.8%) and coagulase**-**negative Staphylococci (9.4%). Also in Gram-negative pathogens, the most resistance was to ampicillin (91.2%), followed by ceftazidime (89.5%). The minimum resistance was against imipenem with prevalence 14.3%.

**Conclusion:**

The combined prevalence of UTI was 32.6%. Gram-negative pathogens especially *E. coli* were the most agents of UTI in Iranian patients who receive kidney transplant. Also, in gram-negative pathogens, the most resistance was to ampicillin that it needs a new strategy for prophylaxis and treatment of UTI after the kidney transplant.

## Introduction

Organ transplant has been identified as a major and selective treatment for patients with a disability and has increased in recent years, and so far, about 11,712 patients are waiting for an organ transplant. The advantages of the organ transplant are; safety in immune-compromised people and cost consuming (1). Due to increased longevity and improved living conditions in receiving transplants, organ donation is rising in the United States and kidney transplant is the most common type. In Iran, the donation of organs of live people and patients with brain death has steadily increased, so that in 2013, the largest number of kidney and liver donations reported in the Middle East (2, 3). One of the organs with the ability to transplantation can mention to the kidney, bone marrow, liver, heart, lung, eye, and in some cases, pancreas and intestines. Immune suppression is the most important factor in the prone of individuals receiving a transplant to infections. Cardiac complications, thrombosis, blood infections, pyelonephritis, hospital infections, intravenous and genital catheter infections are some of the problems encountered by transplants.

Transplant individuals are affected by a variety of viral, protozoal, fungal and bacterial infections (4, 5). Bacterial infections are more common and have a broader form so that they are known to be the most important causes of infection in these patients (6). Bacterial infection was created in the early days after transplantation and according to the reports, 82% of fever after liver transplants, 22–30% of heart transplantation infections, 47% of kidney transplantation infections, 35% of transplantation pancreas infections, and 54% of lung transplantation infections are caused by different bacterial agents (7). Multidrug resistant (MDR) bacteria causing infections in transplant patients, and it has become a fundamental problem in the treatment of infected patients with methicillin-resistant *Staphylococcus aureus* (MRSA), vancomycin-resistant *Enterococci* (VRE), *Enterobacteriaceae* resistant to ciprofloxacin, carbapenem and fluoroquinolones, polymyxin B and ciprofloxacin resistant *Pseudomonas aeruginosa*, carbapenem-resistant *Acinetobacter baumannii*, imipenem and ciprofloxacin resistant Burkholderia (8, 9).

The most common of these transplants is the kidney transplant which its recipients are more prone to infections especially urinary tract infection (UTI). The predisposing factors are; diabetes, immune deficiency and underlying diseases (10). UTI accounts for 60% of transplant infections and has the highest mortality rate. This type of infection occurs during the first 6 months after renal transplantation and based on clinical symptoms; in the first month, asymptomatic bacteriuria in 22–71%, asymptomatic infection in 12–12%, and acute pyelonephritis occur in 6.6% of patients.

The likelihood of the occurrence of pyelonephritis in kidney recipients is high within the first six months post-transplant period and is also associated with the risk of transplant failure and death. Accordingly, it is also associated with the risk of kidney failure and death. For these reasons, acute bacteremia and acute cystitis do not affect individual survival and function of the urinary tract, but acute pyelonephritis can be dangerous in a short time (11).

Despite the advanced surgical techniques, antimicrobial prophylaxis, and new immunosuppressive drugs, UTI is considered as the most important cause of mortality in kidney recipients. In a study, the prevalence of bacterial agents in urinary tract infection in transplant recipients was reported 97%, of which 90% were Gram-negative bacteria and 7% were Gram-positive and *Escherichia coli* was known as the most common cause of infection (71%) (12). In another study conducted by Vidal et al., *E. coli* (55.7%) was identified as the most common bacterial agent causing UTI in recipients of kidney transplantation and other bacteria such as *Pseudomonas aeruginosa* (9.7%), *K. pneumonia* (9.7%), and *Enterococcus* (6.8%) were less commonly detected (13). The frequent and sometimes inappropriate use of antibiotics has led to the emergence of resistant bacteria. More than 70% of bacteria are resistant to at least one antibiotic. Incorrect times, inadequate dosage, or prolonged use of antibiotics are responsible for bacterial resistance and the use of adequate dosage and adequate time in antibiotics application can prevent antibiotic resistance.

Methicillin-resistant *S. aureus*, and vancomycin-resistant *Enterococci* (VRE) isolates is increasing in transplanted individuals and users of intravenous catheters. Therefore, study of prevalence of bacterial agents and their antibiotic resistance is necessary (14). Also, the results of several studies showed that *E. coli* was the most common cause of urinary tract infections in recipients of the kidney (15, 16). The diseases caused by Uropathogenic *E. coli* (UPEC) isolates certainly needs antibacterial therapy; nevertheless, antibiotic-resistant isolates of microorganisms cause more severe diseases for longer periods than their antibiotic-susceptible ones (17). UPEC strains are imposing economic costs for both community and hospital (18). In recent years, the spectrum of antibiotic resistance UTIs agents has changed (19).

Considering the prevalence of bacterial agents in UTI in recipients of kidney and increasing antibiotic resistance, and because there is no detailed systematic review and meta-analysis of infections caused by these bacteria in kidney transplant recipients, we aimed to evaluate the prevalence and antimicrobial resistance of bacterial uropathogens isolated from Iranian kidney transplant recipients.

## Materials and Methods

### Strategy search

We searched according to Prisma protocol(PRISMA, http://www.prisma-statement.org) on the UTI infection, prevalence, occurrence and distribution of bacteria and pattern of antibiotic resistance among Iranian patients who received kidney transplant through online electronic databases including Web of Sciences, PubMed, Scopus and Cochrane Library), and Iranian databases such as Iranmedex (www.iranmedex.com), Scientific Information Database (www.sid.ir), Magiran (www.Magiran.com), Irandoc (www.irandoc.ac.ir) with MeSh terms and text words such as hospital agents, bacterial infection, kidney transplant, post kidney transplant, antibiotic resistance pattern, and Iran. All Published studies in Persian and English languages between the 1990–2017 reporting the prevalence of bacteria and pattern of antibiotic resistance among Iranian patients who received the kidney transplant were studied.

### Inclusion anFd exclusion criteria

The original cross-sectional or cohort references that presenting the prevalence/incidence and distribution of UTI, bacteria, and pattern of antibiotic resistance among Iranian patients who received the kidney transplant were involved in this review. The kinds of literature with sample size of less than 50 deleted of the current study. We excluded review articles, low-quality articles, congress and meeting abstracts, papers stated in languages other than English or Persian, abstract of papers, case report kinds of literature, unrelated papers. Also, this note should be added that to decrease the risk of bias, two researchers independently searched. Articles introduced other than kidney transplants excluded from the present study. Besides, other studies presenting viral, fungal and parasite infections in kidney transplants excluded from our study.

### Data extraction

A special data abstraction form was designed for investigators. The data such as; the first author’s name, time of the study, publication year, the location of study, sample size, UTI prevalence, gender, and mean age were listed in these forms.

### Statistical analysis

Data analysis was performed using Comprehensive meta-analysis software (CMA). Prevalence was reported by 95% confidence intervals (CIs). To calculate the variance in each study for variables (antibiotic resistance, bacteria, UTI) the binomial distribution formula was used. Owing to the large heterogeneity in the prevalence rates in the different studies, Cochrane Q and I^2^ Random Effects Model was used. The I^2^ test was used to evaluate the proportion of statistical heterogeneity and the Q-statistic test was used to explain the degree of heterogeneity. A P-value of less than 0.10 for the Q-test and I^2^ > 50% was considered significant among the articles. For evaluating the possible bias of papers, Egger’s Linear Regression Test was used (20).

## Results

### Literature search and study descriptions

**Fig. 1:**
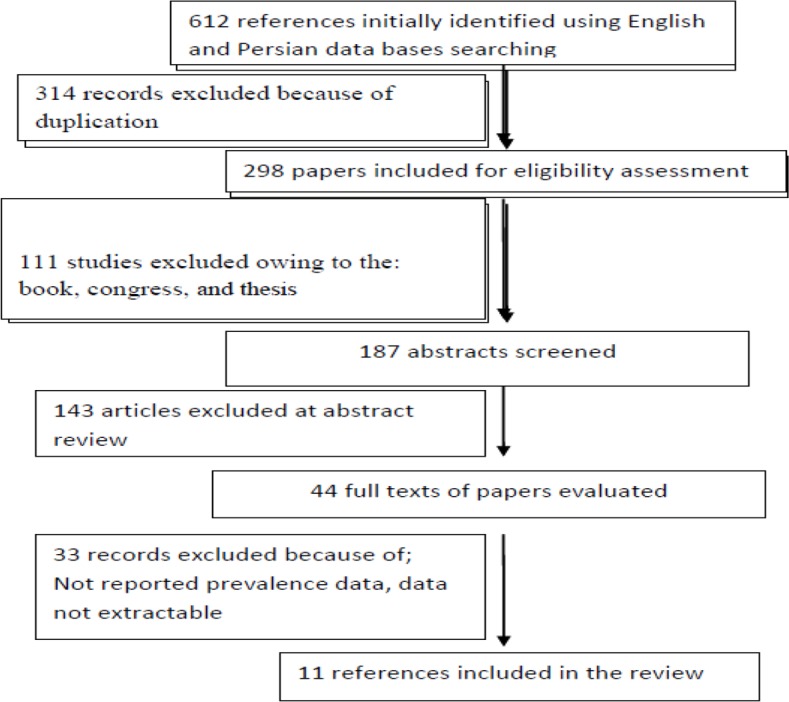
Flow chart of process was used for selecting the studies included in the current study

**Fig. 2:**
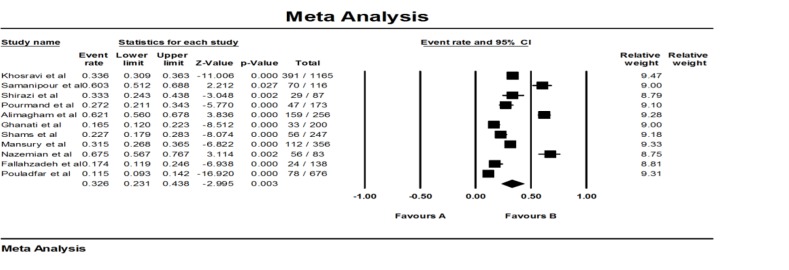
Forest plot of the meta-analysis on prevalence of UTI among Iranian patients who received kidney transplant

The literature search process is described in Fig.1. Briefly, a total of 612 studies, only 11 met eligible inclusion criteria. The features of records enrolled in this review are abstracted in Table 1. The total sample size of the selected studies was 3497 kidney transplant patients. The Prevalence of UTI among kidney transplant patients varied from 11.7% to 67.5% (Table 1, Fig. 2).

**Table 1:** Characteristics of enrolled studies for systematic and meta-analysis

***Study***	***Time of study***	***Publication***	***Location***	***Sample size***	***UTI prevalence***	***Gender (%)***		***Mean Age***
						**Female**	**Male**	
Khosravi et al(41)	2009–2012	2014	Golestan and Ahvaz	1165	32.6	34.8	65.2	39.6 ± 2
Samanipour et al(14)	2013–2014	2015	Tehran	116	60.3	30	70	41.3±13.3
Shirazi et al(42)	1991–1996	2005	Tehran	87	33.3	34.4	65.6	-
Pourmand et al(43)	2011–2012	2012	Tehran	173	27.2	39.3	61.7	40.8 ± 14
Alimagham et al(44)	1993–1997	2002	Tehran	256	62.1	30	70	20–70
Kian Ghanati et al(45)	2009–2010	2012	Tehran	200	16.5	-	-	10–70
Shams et al(16)	2012–2014	2016	Mashhad	247	22.7	40.8	59.2	34.9 ±13.8
Mansury et al(27)	2013–2015	2017	Mashhad	356	31.5	42.1	57.9	-
Nazemian et al(46)	1998–2002	2007	Mashhad	83	67.5	24	76	50–66
Fallahzadeh et al(47)	1990–2008	2011	Shiraz	138	17.4	42.7	57.3	13.6 ± 3.5
Pouladfar et al(40)	2012–2013	2015	Shiraz	676	11.7	50	50	5–87

Studies were reported from North (Golestan province, N=1), South (Shiraz and Ahvaz provinces, N=2), northeast (Mashhad, N=3) and most of those from Center (Tehran, N=5).

Also, 37% and 63% of patients respectively were female and male with a mean age of 5–87 years. Most of the patients with UTI had fever, dysuria, urinary frequency, abdominal pain, nocturia, change in the color and smell of urine. Also, in most cases, the UTI infection occurs after 3 months of receiving the transplant. Asymptomatic UTI was observed in one of the studies. Also, all studies included in this review were used of Kirby Bauer disk diffusion method for assessment of antibiotic susceptibility.

### Overall effects

Of total 11 papers were entered in the current study, based on the results of the heterogeneity test, studies had the heterogeneity (Q2 = 332.2, I2 = 96.9, *P =*0.003). For this reason, to combine the prevalence of UTI, the random effect model was used. The overall prevalence of UTI in recipients of kidney among Iranian patients was 32.6% (23.1–43.8%) (Table 2).

**Table 2:** Subgroups meta-analysis based on the most common bacteria involved in UTI patients

***Subgroups***	***Number of study***	***Random model***			***Heterogeneity test***		***Egger’s test***		
		***Bacteria prevalence (95% CI) (%)***	**Z**	**P**	**P**	**Q**	**I^2^**	**t**	**P**
Overall effects	11	32.6(23.1,43.8)	2.9	0.003	<0.001	332.2	96.9	0.07	0.94
*E. coli*	10	41.3(34.2–48.7)	2.2	0.022	<0.001	56.8	93.3	1.4	0.19
*Enterococcus spp.*	8	9.8(4.3–2.07)	5	0.000	0.00	93	91.3	4.8	0.000
*Klebsiella*	7	11.9(6.8–20)	6.3	0.000	0.000	30.3	80.4	2.6	0.047
*Coagulase negative Staph*	6	9.4(4.8–17.4)	6.2	0.000	0.000	27.7	81.9	0.03	0.97
*Streptococcus*	5	4.9(1.2–18.3)	3.9	0.000	0.000	52.2	92.3	1.5	0.22
*S. aureus*	6	5.9(2.9–11.6)	7.3	0.000	0.003	17.8	72	1.1	0.32
*P. aeruginosa*	7	10(7.2–13.8)	11.8	0.000	0.065	11.8	49.4	2.4	0.04
*Acinetobacter*	4	1.8(0. 9–3.8)	10.4	0.000	0.06	7	57.7	1.9	0.18
*Other*	10	11.4(5.3–22.7)	4.8	0.000	0.00	115	92.1	2.7	0.02

**Fig. 3:**
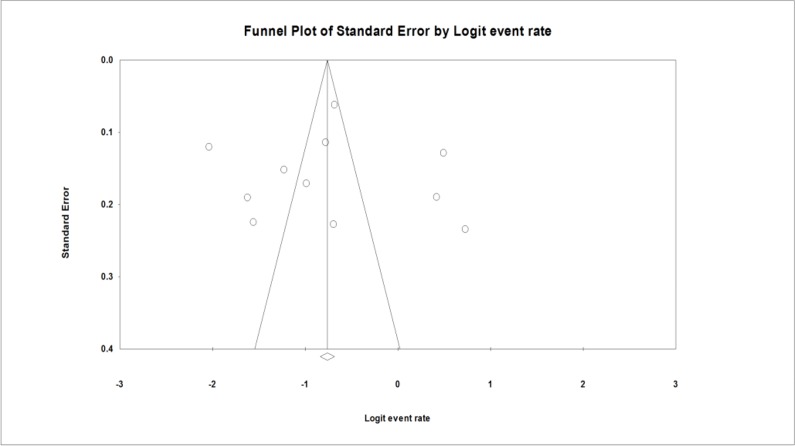
Funnel plot for meta-analysis on prevalence of UTI among Iranian patients who received kidney transplant

The funnel plot was used for assessing publications bias (Fig. 3). In regards to the prevalence of UTI, and owing to the asymmetrical distribution of studies, probably bias was present in the current study, but Egger weighted regression analysis did not confirm this matter (*P =* 0.94).

According to the subgroups analysis, among Gram-negative causing UTI (Table 3), *E. coli* was the most dominant followed by *Klebsiella* with prevalence 41.3% (34.2–48.7), and 11.9% (6.8–20), respectively. Also, amongst Gram-positive bacteria, the highest prevalence was related to *Enterococcus* spp. 9.8% (4.3–2.07), and coagulase-negative staphylococci (CoNS) 9.4% (4.8–17.4). Subgroups meta-analysis of antibiotic resistance for Gram*-*negative recovered of UTI among Iranian patients who received kidney transplant showed the most resistance to ampicillin 91.2% (81.1, 96.2), followed by ceftazidime 89.5% (44.4, 98.9). Resistance to all three antibiotics (carbenicillin, erythromycin and cephalexin) was 86.3% (52.8, 97.2). The minimum resistance was to imipenem with a prevalence of 14.3% (5, 34.7).

**Table 3:** Subgroups meta-analysis of antibiotic resistance for gram negative recovered of UTI among Iranian patients received kidney transplant

***Subgroups***	***Number of study***	***Random model***			***Heterogeneity test***		***Egger’s test***		

		***Resistance rate (95% CI) (%)***	**Z**	**P**	**P**	**Q**	**I^2^**	**t**	**P**
Amikacin	4	40.4(36.4, 44.5)	4.5	0.00	0.4	6.4	0.00	1	0.3
Nitrofurantoin	5	40.9(27.8,55.5)	1.2	0.2	0.00	87.6	87.4	0.5	0.6
Cotrimoxazole	9	70.6(57.3, 81.1)	2.9	0.003	0.00	28.6	72	1.1	0.28
Cephalotin	6	60.8(50.7,70.1)	2.09	0.036	0.2	6.3	21.3	0.8	0.4
Gentamicin	9	51.5(44,51.9)	0.39	0.69	0.27	9.9	19.2	0.1	0.8
Ceftriaxon	3	68.1(44.3,85.2)	1.5	0.13	0.001	14.5	86.2	0.8	0.54
Nalidixic acid	8	56.3(38.2,73)	0.6	0.49	0.00	29.2	76	0.4	0.6
Cefixime	3	66(47.3, 80.8)	1.6	0.09	0.001	13.5	85.2	0.6	0.6
Ciprofloxacin	8	54.5(32,75.3)	0.37	0.7	0.00	74	09	1.3	0.2
Tetracycline	8	60.7(40.3,77.9)	1.02	0.3	0.00	39.4	82.2	1.8	0.1
Pipracillin	3	45.2(14.9,79.6)	0.2	0.8	0.00	24.8	91.9	0.09	0.93
Imipenem	3	14.3(5,34.7)	3	0.002	0.00	50.6	90.1	3.5	0.02
Ceftazidime	3	89.5(44.4,98.9)	1.7	0.07	0.06	5.5	63.6	1	0.4
Ampicillin-Sulbactam	1	37.5(12.5,71.5)	0.6	0.4	1	0.00	0.00	-	-
Piperaciline-tazobactame	2	17.4(6.2, 40.4)	2.6	0.009	0.9	0.36	0.00	-	-
Chloramphenicol	6	42.7(31.4,54.7)	1.1	0.23	0.06	10.5	52.3	0.33	0.75
Amoxicillin	4	69(32.9,91)	1	0.31	0.2	4.2	29.1	11.3	0.007
Tobramycin	3	78.7(42.9, 94.8)	1.6	0.1	0.7	0.4	0.00	3	0.20
Kanamycin	3	56.7(13.7,91.5)	0.24	0.80	0.1	3.4	42.7	0.2	0.8
Ampicillin	2	91.2(81.1,96.2)	5.1	0.00	0.2	1.5	36.1	-	-
Ertapenem	2	17.2(9, 30.4)	4.1	0.00	0.1	1.8	47	-	-
Polymyxin B	2	26(5.1,69.8)	1	0.2	0.64	0.21	0.00	-	-
Erythromycin	3	86.3(52.8,97.2)	2	0.03	0.9	0.03	0.00	-	-
Kanamycin	3	62.7(12.9, 95.2)	0.4	0.6	0.1	4	50.5	0.46	0.72
Polymyxin B	3	46(10.7,85.9)	0.1	0.8	0.2	3	33.4	0.23	0.85
Aztreonam	2	53.5(30.6,75)	0.2	0.7	0.22	5.2	80.8	-	-
Cephalexin	3	86.3(52.8,97.2)	2	0.037	0.9	0.32	0.00	-	-
Carbenicillin	3	86.3(52.8,97.2)	2	0.037	0.9	0.32	0.00	-	-

As shown in Table 4, subgroups meta-analysis of antibiotic resistance for Gram*-*positive isolated of UTI among Iranian patients who received the kidney transplant, the most resistance was to cephalexin with prevalence 80.3% (50.4, 94.2), followed by amoxicillin with prevalence 74.3 %(48.1, 90). The lowest resistance was to polymyxin B with a prevalence of 11.6% (2.3, 41.7). Subgroups meta-analysis of antibiotic resistance for *E. coli* (Table 5) recovered of UTI among Iranian patients who received kidney transplant reported the most resistance to ampicillin 91.2%(81.1,96.2), followed by ceftriaxone with a resistance rate of 87.9% (19.3, 99.5). The least resistance was to imipenem 14.4% (4.2, 39.2), followed by piperacillin-tazobactam 17.4% (6.2, 40.4).

**Table 4:** Subgroups meta-analysis of antibiotic resistance for gram positive recovered of UTI among Iranian patients received kidney transplant

***Subgroups***	***Number of study***	***Random model***			***Heterogeneity test***		***Egger’s test***		
		***Resistance rate (95% CI) (%)***	**Z**	**P**	**P**	**Q**	**I^2^**	**t**	**P**
Amikacin	4	69.4(45.9,85.8)	1.6	0.1	0.4	2.3	0.00	0.16	0.8
Nitrofurantoin	4	35.8(11.3,70.9)	0.7	0.4	0.1	3.7	46	0.1	0.9
Cotrimoxazole	4	45.9(26.1,67.1)	0.3	0.7	0.4	1.8	0.00	0.6	0.6
Cephalotin	4	52.2(30.5,73.1)	0.1	0.8	0.3	2.1	6.2	2.1	0.27
Gentamicin	5	64.4(36.6,85)	1	0.3	0.1	5	40.7	1.1	0.38
Nalidixic Acid	4	45.3(26.1,66)	0.4	0.6	0.7	0.6	0.00	5.3	0.11
Tetracycline	4	48.6(7.1,92.1)	0.04	0.9	0.03	6.8	70.7	0.3	0.7
Amoxicillin	4	74.3(48.1,90)	1.8	0.06	0.32	2.2	12	1.8	0.3
Tobramycin	3	60.2(34.1,81.6)	0.7	0.4	0.5	1	0.00	0.7	0.58
Chloramphenicol	4	67(44.6,83.7)	1.4	0.1	0.5	1.2	0.00	0.2	0.8
Kanamycin	3	71.5(41.6,89.8)	1.4	0.15	0.46	0.45	0.00	-	-
Polymyxin B	3	11.6(2.3,41.7)	2.3	0.019	0.9	0.005	0.00	-	-
Erythromycin	3	71.5(41.6,89.8)	1.4	0.15	0.4	0.5	0.00	-	-
Kanamycin	3	71.5(41.6,89.8)	1.4	0.15	0.4	0.5	0.00	-	-
Cephalexin	3	80.3(50.4,94.2)	1.9	0.04	0.6	0.1	0.00	-	-
Carbenicillin	3	75(32.8,94.9)	1.1	0.23	3.3	0.19	39.5	0.08	0.9

**Table 5:** Subgroups meta-analysis of antibiotic resistance for *E. coli* isolated of UTI among Iranian patients received kidney transplant

***Subgroups***	***Number of study***	***Random model***			***Heterogeneity test***		***Egger’s test***		
		***Resistance rate (95% CI) (%)***	**Z**	**P**	**P**	**Q**	**I^2^**	**t**	**P**
Amikacin	4	38.4(27.8.50.3)	1.9	0.056	0.19	4.7	36.4	0.62	0.59
Nitrofurantoin	5	21(16.8,26)	9.3	0.00	0.42	3.8	0.00	1.1	0.3
Cotrimoxazole	7	73(56.1,85.2)	2.6	0.009	0.001	22	77.2	0.9	0.4
Cephalotin	3	63.6(56.6,70)	3.7	0.00	0.91	0.012	0.00	-	-
Gentamicin	5	53(47.1,58.9)	0.99	0.32	0.4	3.7	0.00	0.08	0.9
Ceftriaxone	2	87.9(19.3,99.5)	1.1	0.25	0.00	11.6	91.4	-	-
Nalidixic acid	4	68.4(34.4,89.9)	1	0.28	0.00	25.5	88.2	1	0.4
Cefixime	2	74.2(43,91.6)	1.5	0.12	0.00	9.9	89.9	-	-
Ciprofloxacin	5	61(23.6,88.8)	0.54	0.58	75.6	0.00	94.7	1.1	0.33
Tetracycline	4	66(30,89.8)	0.86	0.38	0.00	35.5	91.5	1.5	0.27
Pipracillin	2	66.7(24.2,92.6)	0.74	0.45	0.011	5.9	83.1	-	-
Imipenem	5	14.4(4.2,39.2)	2.6	0.009	38.1	0.00	89.5	3.1	0.05
Ceftazidime	2	83.6(1.9,99.9)	0.57	0.56	0.001	12	91.7	-	-
Piperaciline-tazobactame	2	17.4(6.2,40.4)	2.6	0.009	0.33	0.9	0.00	-	-
Chloramphenicol	3	43.4(21.8,67.9)	0.51	0.60	0.1	4.3	54.2	0.039	0.97
Ampicillin	2	91.2(81.1,96.2)	5.1	0.00	0.2	1.5	36.2	-	-

## Discussion

In respect to developments in transplant, survival has resulted in extensive approval of kidney transplantation as the preferred treatment for the patients with End-Stage Renal Disease (ESRD) (21). But UTI is the highest frequent infection subsequently kidney transplantation (22, 23), with a range of 35 to 79% and responsible for around 40–50% of all infectious problems following the kidney transplantation (24). Regarding the studies, most UTIs cases reported through kidney transplantation 1^st^-year post-transplantation (16).

In the present study, the prevalence of UTI among kidney transplant patients varied from 11.7% – 67.5%. This high variation in UTI prevalence likely referred to the varying in the incidences of resistance, postoperative medical care, local outbreaks, different immunosuppressive therapy, diverse diagnostic methods, hygienic statue, quality of nursing services in the general and transplantation surgery wards of hospitals, and administrating proper UTI prophylaxis (16, 24).

The overall prevalence of UTI among Iranian patients who receive kidney transplant was high up to 32% (23.1–43.8%). Similar to our findings, several kinds of literature from different regions of the world showed a high rate of UTI (22, 25, 26).

Gram-negative bacteria are accountable for approximately 70% of UTI, particularly *E. coli* and *Klebsiella pneumonia*. As well as, several Gram-positive such as *Enterococcus* spp. and *Staphylococcus* spp*.* are causing infection in patients who receiving kidney transplants (27).

In this study, among Gram-negative pathogens causing UTI, *E. coli* was the most prevalent followed by *Klebsiella* with prevalence 41.3%, and 11.9%, respectively.

Also, amongst Gram-positive bacteria, the highest prevalence was related to *Enterococcus* spp. 9.8%, and coagulase-negative staphylococci (CoNS) 9.4%. In agreement with our results, a cohort study conducted by Johannes Korth investigate the antibacterial susceptibility of Gram-negative urinary pathogens after kidney transplantation from 2009 to 2012 on 15.741 urine samples were acquired from 859 patients at the Transplant Outpatient Clinic of the University Hospital Essen, Germany. They reported that the most common discovered Gram-negative microbes were *E. coli*, followed by *Klebsiella* spp. and *P. aeruginosa* with prevalence 37%, 8%, and 4.5%, respectively (22). According to the previous studies, *E. coli*, *Enterococcus*, *Staphylococcus*, and *Klebsiella* were the most frequent (28, 29). Several studies in different years from various areas of the world are inconsistent with our findings. They showed the *E. coli* (among Gram-negative*)*, and *Enterococcus* (among Gram-positive) as the major microorganisms recovered from UTI in kidney transplant patients (12, 30).

Of course, contrary to our study, in some studies, other bacteria have been identified as the common cause of urinary tract infections. For example, *Enterobacter cloacae*, and *Klebsiella spp.* were reported as the most causes of post-transplant UTIs, respectively (31).

Prophylaxis with antibiotics is one of the important ways to prevent infections after the kidney transplant. The presence of antibiotic-resistant isolates can cause an increase in the mortality, longer hospital hospitalization, and imposing the higher hospital costs on the patients and healthcare systems than similar infections are caused by antibiotic-susceptible strains (32).

The standard therapy to prevent UTI and other infections after the kidney transplant in most health care settings is the use of Co-trimoxazole (TMP/SMX)(33). Also, the effectiveness of ciprofloxacin has confirmed (34). By contrast, in individuals who have an allergy to mentioned antibiotics, nitrofurantoin is used as prophylaxis (35). Moreover, nitrofurantoin is an effective antibiotic for UTI produced by ESBL producing *E. coli* (36).

In the present study, the resistance rate of Gram-negative bacteria, especially *E. coli* as the most common microorganism recovered from UTI to cotrimoxazole was more than 70%, and to ciprofloxacin was higher than 50%. This issue showing the existence of high resistance to these main antibiotics used in the treatment of UTI among Iranian who received kidney transplant, this possibly attributed to the overuse of these antibiotics which can result in restricted drug choice for the treatment of these infections (37). Also, the resistance rate to nitrofurantoin in Gram-negative organisms was 40.9%, Gram-positive bacteria (35.8%), and *E. coli* (21%). Subgroups meta-analysis of antibiotic resistance for Gram*-*negative recovered of UTI among Iranian patients who received the kidney transplant showed the most resistance to ampicillin (91.2%) followed by ceftazidime (89.5%).

Compatible with our findings, a study conducted by Korth reported that the resistance of Gram-negative isolates to trimethoprimsulfamethoxazole, ciprofloxacin, and ceftazidime increased considerably (22). In a study from México, 22% and 33% of strains tested chiefly Gram-negative isolates respectively, were resistant to ciprofloxacin and ampicillin (30). Of course, this resistance in comparison with our results obtained from the present study was relatively lower.

As well as, another one from Turkey showed resistance rates were 59.4%, 85.7%, 40.7%, and 36.6%, of resistance to ciprofloxacin, cotrimoxazole, ceftriaxone, and gentamicin, respectively (38), which it is in line with our study, too.

According to the results, antibiotic resistance for *E. coli* isolated from UTI among Iranian patients who received kidney transplants reported the most resistance to ampicillin 91.2%, followed by ceftriaxone with a resistance rate of 87.9%. In contrast to the current findings, a study conducted by Kamath et al. in Poland, showed that about 90% of Gram-negative strains were susceptible to ceftriaxone and ceftazidime (39). This high sensitivity refers to the proper use of antibiotics used in this renal transplant center (30).

The most susceptibility in Gram-negative bacteria was observed against Imipenem, and also, in Gram-positive microorganisms, the most effective antibiotic was Polymyxin B. This low resistance probably came back to the low usage of those in kidney transplant settings of Iran.

Undeniably, in this review, the resistance rates of the isolates were high to fluoroquinolones, third-generation cephalosporins, and aminoglycosides. This high resistance results from antibiotic selection pressure and extensive use in kidney transplant patients (40). Therefore none of the mentioned antibiotics except imipenem and piperacillin-tazobactam would be suitable for antibiotic therapy of UTI in patients who received the kidney transplant through hospitalization after kidney transplant.

UTI post-renal transplantation has a high influence on the transplant result. So, the best strategies should be applied to decrease antibiotic resistance and reinforce rational antibiotic treatment. It should be pointed out that the periodic assessment of antibacterial profiles of bacteria related to UTI and reevaluation of the efficiency of antibiotic prophylaxis for the prevention of UTI in patients who receive the kidney transplant is required. An antimicrobial susceptibility should be considered instead of empiric therapy to prevent antibiotic resistance and select the best antibiotic for treatment.

## Conclusion

The combined prevalence of UTI was 32.6%. Gram-negative pathogens especially *E. coli* were the most agents of UTI in Iranian patients who received kidney transplant. Also, in Gram-negative pathogens, the most resistance was to ampicillin that it needs a new strategy for prophylaxis and treatment of UTI after the kidney transplant.

## Ethical considerations

Ethical issues (Including plagiarism, informed consent, misconduct, data fabrication and/or falsification, double publication and/or submission, redundancy, etc.) have been completely observed by the authors.
